# Physician reported incidence of early and late Lyme borreliosis

**DOI:** 10.1186/s13071-015-0777-6

**Published:** 2015-03-15

**Authors:** Agnetha Hofhuis, Margriet Harms, Sita Bennema, Cees C van den Wijngaard, Wilfrid van Pelt

**Affiliations:** Epidemiology and Surveillance Unit, Centre for Infectious Disease Control Netherlands, National Institute for Public Health and the Environment (RIVM), Bilthoven, the Netherlands

**Keywords:** Lyme borreliosis, *Borrelia burgdorferi*, Incidence, Medical diagnoses

## Abstract

**Background:**

Lyme borreliosis is the most common vector-borne disease in Europe and North America. The objective of this study is to estimate the incidence of tick bites and Lyme borreliosis, representative of our entire country, including erythema migrans, disseminated Lyme borreliosis and persisting symptoms attributed to Lyme borreliosis.

**Methods:**

A questionnaire on clinical diagnoses of Lyme borreliosis was sent to all GPs, company physicians, and medical specialists. To adjust for possible misclassification and telescoping bias, we sent additional questionnaires to categorize reported cases according to likelihood of the diagnosis and to exclude cases diagnosed outside the target period.

**Results:**

Adjusted annual incidence rate for disseminated Lyme borreliosis was 7.7 GP reports per 100,000 inhabitants, and for persisting symptoms attributed to Lyme borreliosis was 5.5 GP reports per 100,000 inhabitants, i.e. approximately 1,300 and 900 cases respectively. GP consultations for tick bites and erythema migrans diagnoses were 495 and 132 per 100,000 inhabitants, respectively, i.e. 82,000 and 22,000 cases in 2010.

**Conclusions:**

This is the first reported nationwide physician survey on the incidence of tick bites and the whole range of manifestations of Lyme borreliosis, including persisting symptoms attributed to Lyme borreliosis. This is crucial for complete assessment of the public health impact of Lyme borreliosis.

**Electronic supplementary material:**

The online version of this article (doi:10.1186/s13071-015-0777-6) contains supplementary material, which is available to authorized users.

## Background

Lyme borreliosis is an infectious disease caused by *Borrelia burgdorferi* sensu lato species, and transmitted through tick bites. The disease most commonly manifests as erythema migrans, a slowly expanding skin lesion indicating early localized infection. If the infection spreads to other tissues and organs, it can cause disseminated Lyme borreliosis such as Lyme neuroborreliosis, Lyme arthritis, borrelial lymphocytoma, acrodermatitis chronica atrophicans, Lyme carditis or ocular manifestations. Occasionally, symptoms persist after treatment [1]. Marked increases in the incidence of Lyme borreliosis have been reported over the past decades in several European countries [2-4] and in North America [5,6]. Assessment of the disease burden of Lyme borreliosis requires incidence estimates of all disease outcomes of Lyme borreliosis, ranging from the most common relatively mild early manifestation erythema migrans to the more severe disseminated Lyme borreliosis manifestations, and even persisting symptoms attributed to Lyme borreliosis. However, few incidence estimates from European countries cover all disease expressions of Lyme borreliosis within one surveillance system or survey, representative for a whole country [1-4,7].

Underreporting and misclassification are common to all surveillance systems, and if only high-risk regions are studied, data collections are not representative for whole countries. Particularly with Lyme borreliosis, surveillance is complicated due to the non-specific nature of some disease expressions and the pitfalls of laboratory diagnostics [1-4]. The majority of European countries, including the Netherlands, have not made Lyme borreliosis mandatorily notifiable [2-4]. Most countrywide incidence estimates of Lyme borreliosis in Europe are based on passive reporting laboratory surveillance, using the available details of patients with positive laboratory tests. Unfortunately, erythema migrans is heavily underreported with laboratory surveillance for Lyme borreliosis, because erythema migrans cases are seronegative at presentation, and serologic testing is not routinely requested for erythema migrans. The other manifestations of Lyme borreliosis are overreported with laboratory surveillance, due to seropositivity linked to past exposure [1-4]. Other frequently applied approaches to collecting data on the occurrence of Lyme borreliosis and the relative frequency of its disease manifestations include monitoring of hospital in- and out-patient diagnoses, and physician surveys [8-14]. However none of the published incidence estimations represent the whole country, with exception of two published surveys in France [8,9], based on their GP sentinel surveillance network (a representative selection of all GP’s) and discharge reports of all hospitals.

In the Netherlands, periodic nationwide cross-sectional retrospective studies among general practitioners (GPs) have revealed a continuing and strong increase in GP consultations for tick bites and erythema migrans between 1994 and 2009. The incidence of tick bite consultations increased linearly from 191 per 100,000 in 1994 to 564 per 100,000 inhabitants in 2009, and concurrently the incidence of erythema migrans diagnoses increased from 39 to 134 per 100,000 inhabitants [15-18]. Apart from these nationwide accurate incidence rates for tick bites and erythema migrans over time, the incidence of other manifestations of Lyme borreliosis remained unknown. Furthermore, reports on the occurrence of Lyme borreliosis have not yet touched on the relative occurrence of persisting symptoms attributed to Lyme borreliosis [1-4]. Especially such long-term persisting conditions, with sometimes disabling symptoms, and these can have a great impact on the disease burden and cost-of-illness [1,19].

The objective of the current study is to estimate the incidence of tick bites and the whole range of disease expressions of Lyme borreliosis, including erythema migrans, disseminated Lyme borreliosis and persisting symptoms attributed to Lyme borreliosis. To achieve this, we repeated our earlier nationwide GP surveys, this time including all manifestations of Lyme borreliosis, surveying all GPs, company physicians, and medical specialists who might be involved in the diagnosis of Lyme borreliosis.

## Methods

To rapidly assess a nationwide representative incidence rate for Lyme borreliosis, we performed a two-step approach. Firstly, a broad inquiry was required to also detect the less common disease manifestations. We sent a two-page retrospective questionnaire to all physicians possibly involved in the diagnosis and treatment of Lyme borreliosis in our country: 9178 GPs, 1321 company physicians (i.e. physicians to employee groups, vocational physicians from the private sector), and 5374 medical specialists in hospitals including neurologists, dermatologists, cardiologists, pediatricians, rheumatologists, internists and ophthalmologists. Questionnaires were sent and returned by the postal service. A reminder was sent to non-responding physicians. The questionnaire inquired about consultations for tick bites and diagnosed erythema migrans in 2010. For the less common manifestations of disseminated Lyme borreliosis and persisting symptoms attributed to Lyme borreliosis, we requested clinical diagnoses for the two-year period of 2009–2010. The inquired manifestations of disseminated Lyme borreliosis included borrelial lymphocytoma, acrodermatitis chronica atrophicans, Lyme neuroborreliosis, Lyme arthritis, Lyme carditis and ocular manifestations, and diagnoses of persisting symptoms attributed to Lyme borreliosis, including Lyme encephalopathy and persisting symptoms after treatment for Lyme borreliosis.

As every person in the Netherlands is registered with only one GP, we used the practice populations of reporting GPs to calculate incidence rates and national estimates of total numbers among the population of the Netherlands. As company physicians and medical specialists do not have a clearly defined patient population at risk, their questionnaires were only used for proportional comparison of reported numbers of tick bites and Lyme borreliosis. Physician responses to the questions on consultations for tick bites, erythema migrans diagnoses, and size of practice population were pre-coded in categories to which values were assigned based on the best fit of an assumed underlying negative binomial distribution. Incidence rates for the three categories of Lyme borreliosis were estimated per 100,000 GP practice population in 2010, and bootstrap 95% confidence intervals (95%CI) for the incidence rates were calculated by resampling of the GP reports 10,000 times. Bootstrap resampling was required, as the 95%CI for incidence rates should reflect the number of reporting GPs and each GP practice population combined, instead of the size of the practice populations over all reporting GPs. We screened questionnaires of physicians that reported the top 10% highest incidence of Lyme borreliosis, and excluded questionnaires with clearly deviating answers or unsatisfactory internal consistency (e.g. if a GP reported high numbers of uncommon disease manifestations such as Lyme carditis or ocular manifestations, without reporting common disease manifestations such as erythema migrans, Lyme neuroborreliosis or Lyme arthritis).

To establish case definitions, we adapted the clinical case definitions proposed by Stanek *et al*. [6] with input from physicians of each medical specialism included in the survey, recommended to us by their national associations and input from the Dutch national patients’ association for Lyme borreliosis. Additionally, we designed case definitions for Lyme encephalopathy and persisting symptoms after treatment for Lyme borreliosis. These were included to ensure inclusion of all diagnoses used in GP practice, although these persisting symptoms were analyzed as one category, because there are no clear criteria that distinguish between them. To achieve a high GP response rate, we inquired about clinical diagnoses of Lyme borreliosis in the first step of our two-step approach (see Table 1), not asking the GP to look into laboratory diagnostics that had been ordered and judged by medical specialists in hospitals. Instead, the laboratory diagnostics were verified in the second part of our two-step approach: validation of the GP reports, to verify and adjust the crude incidence rates of disseminated Lyme borreliosis and persisting symptoms attributed to Lyme borreliosis. For this validation we used additional questionnaires, inquiring detailed information on diagnostic criteria. GPs reporting one or two cases of Lyme borreliosis received a questionnaire (see Additional file 1) with regard to applied diagnostic criteria, clinical symptoms, anamnestic tick bites or other diagnoses of Lyme borreliosis, the year of diagnosis with Lyme borreliosis, whether the diagnosis was made by the GP or a medical specialist, treatment, recovery, differential diagnoses and how these were ruled out. GPs reporting more than two cases of Lyme borreliosis received a short questionnaire (see Additional file 2) on general criteria for diagnosis, inquiring whether a medical specialist is normally consulted, and whether laboratory outcomes, clinical symptoms, and anamnestic tick bites or the presence of other (earlier) Lyme borreliosis manifestations are taken into account.

**Table 1:** Clinical case definitions for Lyme borreliosis, mainly* adapted from Stanek ***et al*** **. [** 1 **]**

Erythema migrans	Expanding red or bluish-red patch (= > 5 cm in diameter), with or without central clearing. Advancing edge typically distinct, often intensely coloured, not markedly elevated. If <5 cm in diameter a history of tick-bite, a delay in appearance (after the tick bite) of at least 2 days and an expanding rash at the site of the tick-bite is required.
**Disseminated lyme borreliosis**	
Borrelial lymphocytoma	Painless bluish-red nodule or plaque, usually on ear lobe, ear helix, nipple or scrotum. More frequent in children (especially on ear) than in adults.
Acrodermatitis chronica atrophicans	Long-standing red or bluish-red lesions, usually on the extensor surfaces of extremities. Initial doughy swelling. Lesions eventually become atrophic. Possible skin induration and fibroid nodules over bony prominences.
Lyme neuroborreliosis	In adults, mainly meningo-radiculitis, meningitis. Rarely encephalitis, myelitis, *neuritis*, cerebral vasculitis, *Bannwarth’s syndrome: painful radiculitis, lymphocytic meningitis with facial nerve palsies*. In children, mainly meningitis and facial palsy.
Lyme arthritis	Recurrent attacks or persisting objective joint swelling in one or a few large joints. Alternative explanations must be excluded.
Lyme carditis	Acute onset of atrio-ventricular (I–III) conduction disturbances, rhythm disturbances, sometimes myocarditis or pancarditis. Alternative explanations must be excluded.
Ocular manifestations	Conjunctivitis, uveitis, papillitis, episcleritis, keratitis.
**Persisting symptoms attributed to lyme borreliosis**	
*Lyme encephalopathy*	*Chronic brain syndrome attributed to Lyme borreliosis: impaired memory, concentration, word finding, and sleep; increased fatigue, sensory irritability, emotional lability, headache and depression.*
*Persisting symptoms*	*Persisting symptoms attributed to Lyme borreliosis after treatment, with or without active Borrelia infection.*

*Additional case definitions for ‘persisting symptoms attributed to Lyme borreliosis’, which were not proposed by Stanek *et al.* [1], are indicated with *Italic printing* in this table, as well as modifications in the case definition for ‘Lyme neuroborreliosis’.

Considering the complexities in the diagnosis of Lyme borreliosis, we categorized the cases of disseminated Lyme borreliosis according to the likelihood of diagnosis into ‘very likely’, ‘likely’, and ‘possible’ (see Table 2). We labelled GP reports as ‘invalid reports’, when a GP declared to have erroneously marked the Lyme borreliosis case on the questionnaire, or if the diagnosis Lyme borreliosis was abandoned after further diagnostics. For persisting symptoms attributed to Lyme borreliosis we left out the ‘very likely’ category, as this diagnosis is always uncertain [1]. To correct for ‘telescoping bias’ [20], we determined the percentage of cases diagnosed with disseminated Lyme borreliosis within the target period of 2009 and 2010. The target period for diagnosis for persisting symptoms attributed to Lyme borreliosis was extended to 2008–2010, to include the cases who developed Lyme borreliosis in 2008 and were diagnosed with persisting symptoms attributed to Lyme borreliosis in 2009–2010. We adjusted the crude incidence rates of our incidence survey according to the proportion of “very likely” diagnosis for disseminated Lyme borreliosis, and “likely” diagnosis for persisting symptoms attributed to Lyme borreliosis, and according to the proportion of GP reports within the targeted period of diagnosis (2009 and 2010 for disseminated Lyme borreliosis, 2008 to 2010 for persisting symptoms attributed to Lyme). Additionally, we performed a secondary adjustment scenario to estimate the occurrence of all Lyme borreliosis reports, including the less likely reports, because they do contribute to the disease burden and costs for our society as well as the individual patient. In this target period adjustment scenario, only invalid reports were excluded and crude incidence was adjusted according to the proportion of GP reports within the targeted period of diagnosis. Data pre-processing and statistical analyses were performed in SAS version 9.1.3, and in R version 3.0.1. In this study, physicians reported only the number of patients diagnosed with Lyme borreliosis and diagnostic methods used to make these diagnoses. Therefore, the Medical Ethics Review Committee UMC Utrecht declared that the Medical Research Involving Human Subjects Act does not apply to this study (protocol number 14-415/C, letter number WAG/th/14/021989).

**Table 2:** Classification according to likelihood of the diagnosis, and proportion of diagnoses within the targeted period, of validated general practitioner (GP) reported cases of disseminated Lyme borreliosis* and persisting symptoms attributed to Lyme borreliosis**

Likelihood of diagnoses for disseminated Lyme borreliosis*	**(n/N)**	**Percentage (95%CI)**
*Criteria for classification*		
**Very likely diagnosis**	(527/590)	89.3% (86.5%-91.6%)
*- Diagnosed by a medical specialist.*		
*OR*		
*- Diagnosis based on clinical symptoms, laboratory diagnosis and anamnesis.*		
**Likely diagnosis**	(27/590)	4.6% (3.1%-6.7%)
*- Diagnosis based on clinical symptoms and anamnesis.*		
*OR*		
*- Diagnosis based on laboratory diagnosis and anamnesis.*		
**Possible diagnosis**	(22/590)	3.7% (2.4%-5.7%)
*- Diagnosis not made by medical specialist.*		
*- Diagnosis not based on clinical symptoms, laboratory diagnosis and anamnesis.*		
*- Diagnosis not based on clinical symptoms and anamnesis.*		
*- Diagnosis not based on laboratory diagnosis and anamnesis.*		
*OR*		
*- GP reports that the diagnosis is uncertain.*		
*OR*		
*- Patient proposed diagnosis Lyme borreliosis, but GP or medical specialist disagree.*		
*- Diagnosed by an alternative healer.*		
**Invalid report**	(14/590)	2.4% (1.4%-4.1%)
*- GP declared to have erroneously marked the Lyme borreliosis case on the questionnaire.*		
*- The diagnosis Lyme borreliosis was abandoned after further specific diagnostics.*		
**Diagnosed within targeted period**	(89/141)	63.1% (54.6%-70.9%)
*2009 or 2010*		
Likelihood of diagnoses for persisting symptoms attributed to lyme borreliosis**	**(n/N)**	**Percentage (95%CI)**
*Criteria for classification*		
**Likely diagnosis**	(414/544)	76.1% (72.2%-79.6%)
*- Diagnosed by a medical specialist.*		
*OR*		
*- Diagnosis based on clinical symptoms, laboratory diagnosis and anamnesis.*		
**Possible diagnosis**	(106/544)	19.5% (16.3%-23.1%)
*- Diagnosis based on clinical symptoms and anamnesis.*		
*OR*		
*- Diagnosis based on laboratory diagnosis and anamnesis.*		
*OR*		
*- GP reports that the diagnosis is uncertain.*		
*OR*		
*- GP reports that the diagnosis is uncertain.*		
*- Patient proposed diagnosis Lyme borreliosis, but GP or medical specialist disagree.*		
*- Diagnosed by an alternative healer.*		
**Invalid Report**	(24/544)	4.4% (2.9%-6.6%)
*- GP declared to have erroneously marked the Lyme borreliosis case on the questionnaire.*		
*- The diagnosis Lyme borreliosis was abandoned after further specific diagnostics.*		
**Diagnosed within targeted period**	(41/78)	52.6% (41.0%-63.9%)
*2008 or 2010*		

95%CI, 95% confidence interval.

*“Disseminated Lyme borreliosis” includes: borrelial lymphocytoma, acrodermatitis chronica atrophicans, Lyme neuroborreliosis, Lyme arthritis, Lyme carditis, ocular manifestations.

**“Persisting symptoms attributed to Lyme borreliosis” includes: encephalopathy, persisting symptoms after treatment for Lyme borreliosis with or without active *Borrelia* infection.

## Results

**Figure 1:**
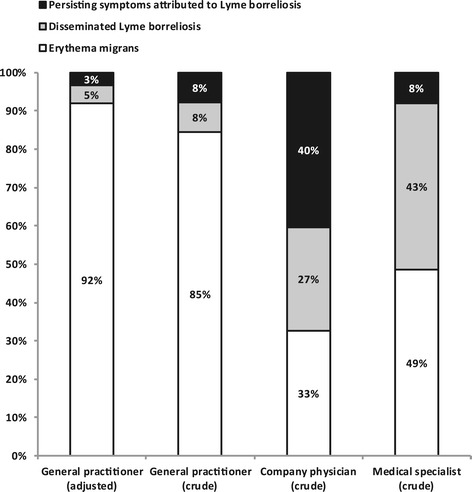
**Lyme borreliosis manifestations as a proportion of all Lyme-related diagnoses, by type of physician.**

Among GPs, the response rate to our questionnaire was 39% (3584 out of 9178), representing a total practice population of 8.7 million persons, which is 53% of the 16.6 million inhabitants of the Netherlands. Among company physicians, the response rate was 30% (391/1321) representing one million employees, which is 14% of our country’s labor force. Among medical specialists, the response was 35% (1860/5347, hospital catchment population unknown). Table 3 shows the crude and adjusted incidence and national numbers, and 95% CI of tick bites and Lyme borreliosis in 2010. The incidence rates of GP consultations for tick bites and erythema migrans diagnoses respectively were 495 (95% CI 478–512) and 132 (95% CI 127–136) per 100,000 inhabitants in 2010. All GPs nationwide saw approximately 82,000 patients with tick bites and 22,000 patients with erythema migrans in 2010. The crude incidence of disseminated Lyme borreliosis was 13.6 (95% CI 12.7-14.5) per 100,000 inhabitants, and for persisting symptoms attributed to Lyme borreliosis we estimated an incidence of 13.7 (95% CI 12.7-14.6) per 100,000 inhabitants. The clinical diagnoses for Lyme borreliosis among GPs were mainly (85%) for erythema migrans (see Figure 1). Of all Lyme-related diagnoses at the GP, 7.5% concerned disseminated Lyme borreliosis, and another 7.5% concerned persisting symptoms attributed to Lyme borreliosis. Erythema migrans is usually diagnosed by GPs, but they still represent 33% of all Lyme borreliosis diagnoses made by company physicians and 49% of all Lyme borreliosis diagnoses made by medical specialists. For the latter, diagnoses of disseminated Lyme borreliosis and persisting symptoms attributed to Lyme borreliosis together represent more than half of their Lyme borreliosis diagnoses. Lyme borreliosis diagnoses by company physicians are predominantly about persisting symptoms, but also include disseminated Lyme borreliosis (Figure 1).

**Table 3:** **Crude and adjusted incidence rates of general practitioner consultations for tick bites and Lyme borreliosis diagnoses per 100,000 inhabitants, and national estimates of the total numbers among the 16.6 million inhabitants of the Netherlands in 2010**

	**Incidence (95% CI) per 100,000**		**National numbers**	
**Crude estimations**				
Tick bites	494.7	(478.1-511.8)	81,997	(79,253 – 84,827)
Erythema migrans	131.5	(127.1-136.0)	21,802	(21,064 – 22,545)
Disseminated Lyme borreliosis*	13.6	(12.7-14.5)	2,250	(2,103 – 2,400)
Persisting symptoms attributed to Lyme borreliosis**	13.7	(12.7-14.6)	2,263	(2,112 – 2,416)
Adjusted estimations***				
Disseminated Lyme borreliosis*	7.7	(7.2-8.2)	1,268	(1,186-1,353)
Persisting symptoms attributed to Lyme borreliosis**	5.5	(5.1-5.8)	905	(845–966)
Target period adjusted estimations****				
Disseminated Lyme borreliosis*	8.4	(7.8-8.9)	1,386	(1,296-1,479)
Persisting symptoms attributed to Lyme borreliosis**	6.9	(6.4-7.3)	1,137	(1,061-1,214)

95%CI, 95% confidence interval.

*“Disseminated Lyme borreliosis” includes: borrelial lymphocytoma, acrodermatitis chronica atrophicans, Lyme neuroborreliosis, Lyme arthritis, Lyme carditis, ocular manifestations.

**“Persisting symptoms attributed to Lyme borreliosis” includes: encephalopathy, persisting symptoms after treatment for Lyme borreliosis with or without active *Borrelia* infection.

***Primary adjustment scenario:

The incidence for disseminated Lyme borreliosis was adjusted according to the proportion of very likely diagnosis, and to the proportion of GP reports within the targeted period of diagnosis 2009 and 2010 (see Table 2).

The incidence for persisting symptoms attributed to Lyme borreliosis was adjusted according to the proportion of likely diagnosis, and to the proportion of GP reports within the targeted period of diagnosis 2008 to 2010 (see Table 2).

****Target period adjustment scenario:

The incidence for disseminated Lyme borreliosis was adjusted according to the proportion of very likely, likely, and possible diagnosis, and to the proportion of GP reports within the targeted period of diagnosis 2009 and 2010 (see Table 2).

The incidence for persisting symptoms attributed to Lyme borreliosis was adjusted according to the proportion of likely diagnosis and possible diagnosis, and to the proportion of GP reports within the targeted period of diagnosis 2008 to 2010 (see Table 2).

The response rate to our validation questionnaire among GPs was 47% (855/1832) for the short questionnaire, and 20% (276/1399) for the long questionnaire. As shown in Table 2, 89.3% of the cases of disseminated Lyme borreliosis satisfied our validation criteria for a very likely diagnosis, 4.6% were categorized as likely, and 3.7% was categorized as possible. For persisting symptoms attributed to Lyme borreliosis, 76.1% of the cases satisfied our criteria for a likely diagnosis and 19.5% was categorized as possible. 2.4% and 4.4% of the reports were categorized as invalid for disseminated Lyme borreliosis and persisting symptoms attributed to Lyme borreliosis, respectively. For disseminated Lyme borreliosis, 63.1% of the cases were diagnosed within the targeted period of 2009 and 2010. For persisting symptoms attributed to Lyme borreliosis, 52.6% of the cases were diagnosed within the targeted period of 2008 to 2010.

**Figure 2:**
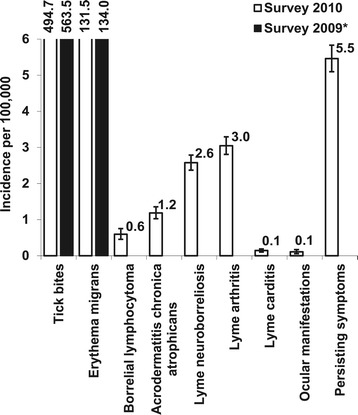
**Adjusted incidence rates of general practitioner consultations for tick bites and Lyme borreliosis diagnoses per 100,000 inhabitants of the Netherlands in 2010.** Vertical bars represent 95% confidence intervals. *For comparison, the incidence of tick bite consultations and diagnoses of erythema migrans diagnoses per 100,000 inhabitants in 2009 was 564 and 134, respectively [18].

We estimated the adjusted annual incidence for a very likely diagnosis of disseminated Lyme borreliosis at 7.7 (95% CI 7.2-8.2) per 100,000 inhabitants in 2009 and 2010. For persisting symptoms attributed to Lyme borreliosis, we estimated the adjusted annual incidence at 5.5 (95% CI 5.1-5.8) per 100,000 inhabitants. Consequently, the national number of patients annually seen by all GPs in 2009 and 2010 was estimated at approximately 1300 patients with disseminated Lyme borreliosis and 900 patients with persisting symptoms attributed to Lyme borreliosis (Table 3). After adjusting, erythema migrans represented 92% of the clinical diagnoses for Lyme borreliosis reported by the GP, while 4.7% of the diagnoses concerned disseminated Lyme borreliosis, and 3.4% concerned persisting symptoms attributed to Lyme borreliosis (see Figure 1). Figure 2 shows the adjusted incidence per Lyme borreliosis manifestation. Lyme arthritis and Lyme neuroborreliosis were the most frequent clinical manifestations of disseminated Lyme borreliosis, with annual incidence rates of 3.0 (95% CI 2.8-3.3) and 2.6 (95% CI 2.4-2.8) per 100,000 inhabitants. Finally, the scenario to estimate the occurrence of all Lyme borreliosis reports including less likely diagnoses, yielded an annual incidence rate of 8.4 diagnoses per 100,000 inhabitants for disseminated Lyme borreliosis, and 6.9 diagnoses per 100,000 inhabitants for persisting symptoms attributed to Lyme borreliosis in 2009 and 2010 (see 95% CI in Table 3).

## Discussion

We report the first incidence estimations of disseminated Lyme borreliosis and persisting symptoms attributed to Lyme borreliosis, based on a short questionnaire with clinical case definitions, sent to all GPs in the Netherlands. Furthermore, this is the first reported physician survey on tick bites and all manifestations of Lyme borreliosis that covers an entire country. The incidence rates of GP consultations for tick bites and erythema migrans diagnoses respectively were 495 and 132 per 100,000 inhabitants in 2010. After validation of the GP reports, we estimated an adjusted annual incidence of 7.7 very likely disseminated Lyme borreliosis diagnoses per 100,000 inhabitants of the Netherlands in 2009 and 2010, and 5.5 likely diagnoses of persisting symptoms attributed to Lyme borreliosis per 100,000 inhabitants in 2009 and 2010 (see Table 3). When less likely diagnoses were included to estimate the occurrence of all Lyme borreliosis reports, the annual incidence rate was 8.4 for disseminated Lyme borreliosis diagnoses per 100,000 inhabitants, and 6.9 diagnoses for persisting symptoms attributed to Lyme borreliosis per 100,000 inhabitants. These estimates reflect all morbidity attributed to Lyme borreliosis by GPs. There is an ongoing debate to what extent past or present *Borrelia* infection actually causes this condition of persisting symptoms attributed to Lyme borreliosis [21]. Nevertheless, about 900 patients consulted their GP in 2010 for such persisting symptoms which their GP attributed to Lyme borreliosis. This calls for further research to the causal mechanisms of this condition – whether or not caused by past or present *Borrelia* infection – to improve clinical care for this substantial patient group.

Not all patients with Lyme borreliosis are correctly diagnosed and treated in practice, which may lead to underestimation of the disease occurrence. Some publications on underreporting of Lyme borreliosis state that the true incidence rate may be two to eight times higher than measured [22,23]. On the other hand, misdiagnosis and misclassification can cause overestimation, which we reduced through quantification of telescoping bias, and validation of the likelihood of diagnosis, based on the applied clinical and laboratory criteria.

We achieved sufficient GP response to account for 53% of the inhabitants of the Netherlands, which is comparable to the response rates of the preceding GP surveys. The incidence rates for tick bites and erythema migrans in 2010 were similar to the estimates from the GP survey in 2009, confirming that the extended survey was consistent with the earlier GP studies. The small differences can be ascribed to the annual fluctuation typically observed in the surveillance of Lyme borreliosis [5,6].

Comparability of incidence rates between countries is poor, due to differences in data collection. Accordingly, divergent incidence rates for Lyme borreliosis have been reported, and only year-to-year comparisons of the incidence rate within countries can be made [2-4]. However, countries can be compared as to the proportional occurrence of clinical manifestations of Lyme borreliosis. As opposed to physician based studies from other countries [8-10,12,14], persisting symptoms attributed to Lyme borreliosis were included in our questionnaire and case definitions. Excluding persisting symptoms attributed to Lyme borreliosis, we observed a proportion of 95% of erythema migrans diagnoses relative to all GP-reported clinical manifestations of Lyme borreliosis. This is in line with the proportions reported in France and Southern and Eastern Germany while in Southern Sweden a lower proportion of 77% was found [8,10,12,14]. In our study, as well as in France, Germany and Sweden, the two most commonly GP-reported disseminated manifestations of Lyme borreliosis were Lyme neuroborreliosis and Lyme arthritis [8-10,12,14]. Borrelial lymphocytoma, acrodermatitis chronica atrophicans, Lyme carditis and ocular manifestations were uncommon in all countries [8-10,12,14].

Comparability between countries is also hampered by differences in health-care systems. Patients in the Netherlands are required to consult a GP to be referred to a medical specialist in a hospital. As a result, erythema migrans is usually diagnosed and treated by GPs, and not by medical specialists. This is illustrated by our relatively low proportion of diagnoses by medical specialists, as compared to GP diagnoses (53% vs 95%), although the proportion of hospital diagnoses of erythema migrans from Northeastern France (60%) [13], and in a group of hospitals surveyed in fifteen European countries (59% of Lyme-related skin manifestations) [11] was only slightly higher. The latter study of Cimmino *et al*. in 1994 included the Netherlands, where six medical specialists reported 42% skin manifestations, mostly erythema migrans.

Considering all GP-reported Lyme borreliosis, 4.7% of the diagnoses concerned disseminated Lyme borreliosis, and 3.4% concerned persisting symptoms attributed to Lyme borreliosis (see Figure 1). Due to the high disease burden and costs associated with disseminated Lyme borreliosis and persisting symptoms attributed to Lyme borreliosis, they will contribute greatly to the public health impact of Lyme borreliosis in our society. Combined with data on disease burden and costs, the annual numbers per disease manifestation estimated in this study can be used to assess the annual disease burden and cost-of-illness of Lyme borreliosis in the Netherlands.

## Conclusion

The current study demonstrates that nationwide representative incidence rates for GP reported tick bites and all manifestations of Lyme borreliosis can be obtained rapidly through a two-step approach of a cross-sectional retrospective questionnaire among physicians, followed by a validation questionnaire to adjust for possible misclassification and telescoping bias. We report the first estimations for the occurrence in the Netherlands of disseminated Lyme borreliosis and of persisting symptoms attributed to Lyme borreliosis, respectively 7.7 and 5.5 GP diagnoses per 100,000 inhabitants. These estimates are crucial for assessment of the public health impact of Lyme borreliosis.

## Additional files

Additional file 1:
**Questionnaire for validation of general practitioner reported diagnoses of Lyme borreliosis.**
Additional file 2:
**Short questionnaire for validation of general practitioner reported diagnoses of Lyme borreliosis.**
