# Erratum to: Physician reported incidence of early and late Lyme borreliosis

**DOI:** 10.1186/s13071-015-0990-3

**Published:** 2015-07-17

**Authors:** Agnetha Hofhuis, Margriet Harms, Sita Bennema, Cees C. van den Wijngaard, Wilfrid van Pelt

**Affiliations:** Epidemiology and Surveillance Unit, Centre for Infectious Disease Control Netherlands, National Institute for Public Health and the Environment (RIVM), Bilthoven, The Netherlands

After the publication of this work [1], we noticed an error in the percentages of “Fig. 1 Lyme borreliosis manifestations as a proportion of all Lyme-related diagnoses, by type of physician”. The percentages of the first horizontal bar “General Practitioner (adjusted)” should be: 90.9 % erythema migrans (instead of 92 %), 5.3 % disseminated Lyme borreliosis, and 3.8 % persisting symptoms attributed to Lyme borreliosis (instead of 3 %).

**Fig. 1:**
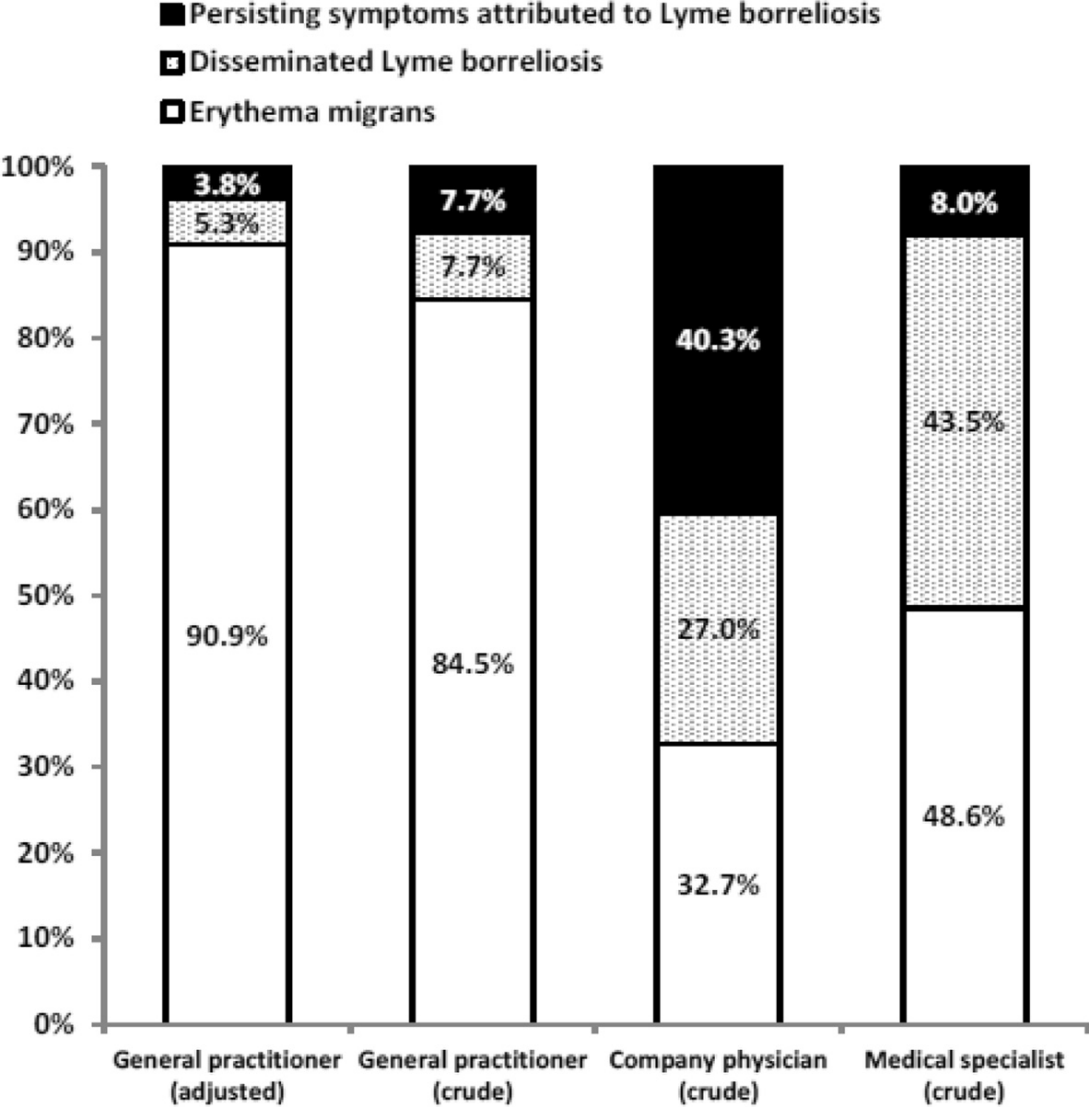
Lyme borreliosis manifestations as a proportion of all Lyme-related diagnoses, by type of physician

Consequently, the corresponding sentence in the results section should be: “After adjusting, erythema migrans represented 90.9 % of the clinical diagnoses for Lyme borreliosis reported by the GP, while 5.3 % of the diagnoses concerned disseminated Lyme borreliosis, and 3.8 % concerned persisting symptoms attributed to Lyme borreliosis (see Fig. 1)”.

And the corresponding sentence in the discussion section should be: “Considering all GP-reported Lyme borreliosis, 5.3 % of the diagnoses concerned disseminated Lyme borreliosis, and 3.8 % concerned persisting symptoms attributed to Lyme borreliosis (see Fig. 1).”
