# Supplementary material for: Physician reported incidence of early and late Lyme borreliosis
Source: Parasit Vectors. 2015 Mar 15;8:161. doi: 10.1186/s13071-015-0777-6 (PMC4363353; doi:10.1186/s13071-015-0777-6)
Supplement: Additional file 1: — Questionnaire for validation of general practitioner reported diagnoses of Lyme borreliosis. [file 13071_2015_777_MOESM1_ESM.docx]

**Additional file 1**

**Questionnaire for validation of general practitioner reported diagnoses of Lyme borreliosis.**

GPs reporting one or two cases of Lyme borreliosis received an extensive questionnaire. For each GP, the topic of each questionnaire was customised to the reported disease manifestations of Lyme borreliosis (indicated as *LB*), among which: borrelial lymphocytoma, acrodermatitis chronica atrophicans, Lyme neuroborreliosis, Lyme arthritis, Lyme carditis, ocular manifestations, Lyme encephalopathy, and persisting symptoms after treatment for Lyme borreliosis.

**Year of birth of the patient:** …………….

**Gender of the patient:** …………….

**What year did the patient develop Lyme borreliosis?** …………….

**Who diagnosed *LB * in this patient?**

❑ I did (the general practitioner)
❑ medical specialist: Name Mr/Mrs ……………. Hospital: ……………. Specialism: …………….

**What was the diagnosis *LB* in this patient based on?** (multiple answers possible):

❑ clinical presentation

❑ laboratory outcomes

❑ unknown

**What clinical symptoms did this patient have?** …………….

**How long did these symptoms exist at the moment of diagnosis?** …………….

**Did the patient’s anamnesis fulfil one of the following characteristics?**

❑ erythema migrans shortly before or during the onset of *LB*

❑ other clinical manifestations of Lyme borreliosis shortly before or during the onset of *LB*

❑ a tick bite, clearly related in time to the onset of symptoms

❑ a tick bite, not related in time to the onset of symptoms

❑ frequent exposure to tick bites

❑ no, none of these characteristics

❑ unknown

**What laboratory diagnostics were performed on what material, and what were the outcomes?** (multiple answers possible):

❑ ELISA / Western Blot on: ……………. , outcome: …………….

❑ PCR on: ……………. , outcome: …………….

❑ culture of *B. burgdorferi* s.l. on: ……………., outcome: …………….

❑ histology on: ……………., outcome: …………….

❑ tests on cerebrospinal fluid:

❑ ELISA / Western Blot on intrathecal fluid, outcome: …………….

❑ pleocytosis, outcome: …………….

❑ total protein concentration, outcome: …………….

❑ glucose concentration, outcome: …………….

❑ other tests (e.g. LTT-MELISA, CD57 expression, C3/C4 levels, etc)? Which test: ……………., outcome: …………….

❑ no laboratory diagnostics performed

❑ laboratory diagnostics unknown

**How was the patient treated?** ……………., dose: ……………., duration: …………….

**Did the patient experience symptoms due to Lyme borreliosis, after the first antibiotic treatment?**

❑ No, the symptoms resolved

❑ Yes, but the symptoms were less severe

❑ Yes, severity of symptoms did not change

❑ Yes, the symptoms worsened

**Which differential diagnoses were considered and how were these excluded?** …………….

**Final remarks and other relevant information:** …………….
