# Supplementary material for: Physician reported incidence of early and late Lyme borreliosis
Source: Parasit Vectors. 2015 Mar 15;8:161. doi: 10.1186/s13071-015-0777-6 (PMC4363353; doi:10.1186/s13071-015-0777-6)
Supplement: Additional file 2: — Short questionnaire for validation of general practitioner reported diagnoses of Lyme borreliosis. [file 13071_2015_777_MOESM2_ESM.doc]

**Additional file 2**

**Short questionnaire for validation of general practitioner reported diagnoses of Lyme borreliosis.**

GPs reporting more than two cases of Lyme borreliosis received a short questionnaire. For each GP, the topic of each questionnaire was customised to the reported disease manifestations of Lyme borreliosis (indicated as *LB*), among which: borrelial lymphocytoma, acrodermatitis chronica atrophicans, Lyme neuroborreliosis, Lyme arthritis, Lyme carditis, ocular manifestations, Lyme encephalopathy, and persisting symptoms after treatment for Lyme borreliosis.

**How do you generally diagnose *LB*?** (multiple answers possible):

❑ I consult / refer the patient to a medical specialist in the hospital

❑ based on clinical presentation

❑ based on laboratory outcomes

❑ otherwise: …………….

❑ unknown

**Which of the following characteristics do you consider for the diagnosis of *LB*?** (multiple answers possible):

❑ erythema migrans shortly before or during the onset of *LB*

❑ other clinical manifestations of Lyme borreliosis shortly before or during the onset of *LB*

❑ a tick bite, clearly related in time to the onset of symptoms

❑ a tick bite, not related in time to the onset of symptoms

❑ frequent exposure to tick bites

❑ no, none of these characteristics

❑ something else: …………….

❑ unknown

**Final remarks and other decisive factors in the diagnosis of *LB*:** …………….
